# The vascularization paradox of non-union formation

**DOI:** 10.1007/s10456-022-09832-x

**Published:** 2022-02-14

**Authors:** Maximilian M. Menger, Matthias W. Laschke, Andreas K. Nussler, Michael D. Menger, Tina Histing

**Affiliations:** 1 Department of Trauma and Reconstructive Surgery, BG Trauma Center Tuebingen, Eberhard Karls University Tuebingen, 72076 Tuebingen, Germany; 2 Institute for Clinical & Experimental Surgery, Saarland University, 66421 Homburg/Saar, Germany; 3 Department of Trauma and Reconstructive Surgery, BG Trauma Center Tuebingen, Siegfried Weller Institute for Trauma Research, Eberhard Karls University Tuebingen, 72076 Tuebingen, Germany

**Keywords:** Non-union, Vascularization, Angiogenesis, Bone healing, VEGF, Bone healing

## Abstract

Despite major research efforts to elucidate mechanisms of non-union formation, failed fracture healing remains a common complication in orthopedic surgery. Adequate vascularization has been recognized as a crucial factor for successful bone regeneration, as newly formed microvessels guarantee the supply of the callus tissue with vital oxygen, nutrients, and growth factors. Accordingly, a vast number of preclinical studies have focused on the development of vascularization strategies to stimulate fracture repair. However, recent evidence suggests that stimulation of blood vessel formation is an oversimplified approach to support bone regeneration. This review discusses the role of vascularization during bone regeneration and delineates a phenomenon, for which we coin the term “the vascularization paradox of non-union-formation”. This view is based on the results of a variety of experimental studies that suggest that the callus tissue of non-unions is indeed densely vascularized and that pro-angiogenic mediators, such as vascular endothelial growth factor, are sufficiently expressed at the facture site. By gaining further insights into the molecular and cellular basis of non-union vascularization, it may be possible to develop more optimized treatment approaches or even prevent the non-union formation in the future.

## Introduction

Despite impressive progress in our understanding of the mechanisms of delayed healing and non-union formation, failed fracture healing still represents a major clinical challenge. Non-unions are defined by the U.S. Federal Drug Administration as ‘failure to achieve union by 9 months since the injury, and for which there has been no signs of healing for 3 months’ [1]. However, others define non-union formation in long bones after a period of 6 months with no radiological sign of fracture healing [2]. In general, the diagnosis of non-union should include both the clinical and radiological examination of the patient [3].

Large segmental bone defects, infections, tumors, and systemic comorbidities as well as mechanical instabilities associated with insufficient osteosynthesis bear a high risk of non-union formation [4–7]. However, in many cases, the cause of fracture healing failure is unclear, and thus, effective treatment strategies are lacking. Accordingly, the failure rate of fracture healing is still up to 10% [8]. Furthermore, non-unions do not only result in significant pain and loss of function with subsequent reduction of quality of life, but additionally cause a substantial economic burden on the health care system [9].

Bone regeneration involves multiple biological and biochemical processes. Among these, vascularization is supposed to be essential for successful fracture healing [10, 11]. Bone is a highly vascularized tissue, which crucially depends on the close spatial and temporal interaction between blood vessels and osteogenic cells to maintain bone development and remodeling [12]. During bone repair, the skeletal vasculature provides vital cells, hormones, and nutrients to the fracture site to allow for callus remodeling from avascular cartilaginous tissue toward mineralized woven bone [13]. Therefore, a considerable number of studies have focused on the application of vascularization strategies to prevent or treat non-union formation. These strategies involve (i) biophysical applications, (ii) systemic pharmacological interventions, and (iii) tissue engineering, including the development of sophisticated scaffold materials, local growth factor delivery systems, cell-based techniques, and surgical vascularization approaches [10].

However, there is evidence that vascularization is only one piece of the puzzle in the much more complex process of bone regeneration. Hence, this review compares and discusses the current literature focusing on the role of vascularization within the complex scenario of bone regeneration, elucidating both, supportive and inhibitory actions of the blood vessel formation on the healing outcome.

### The role of vascularization in bone regeneration

The skeletal vasculature is important for bone development and remodeling as well as for bone regeneration [14, 15]. Previous studies using dye injections and radiomicropraphs for the visualization of blood vessels have provided important fundamental data on the organization of the bone vasculature, particularly in long tubular bones [16, 17]. More recently, a plentitude of technological advancements in immunohistochemical and diagnostic imaging techniques, such as cell type-specific markers as well as confocal or two-photon microscopy and microcomputed tomography (µCT) with three-dimensional imaging reconstruction, have markedly improved our understanding of the morphology and specialized functions of the bone vasculature [18–21].

**Fig. 1:**
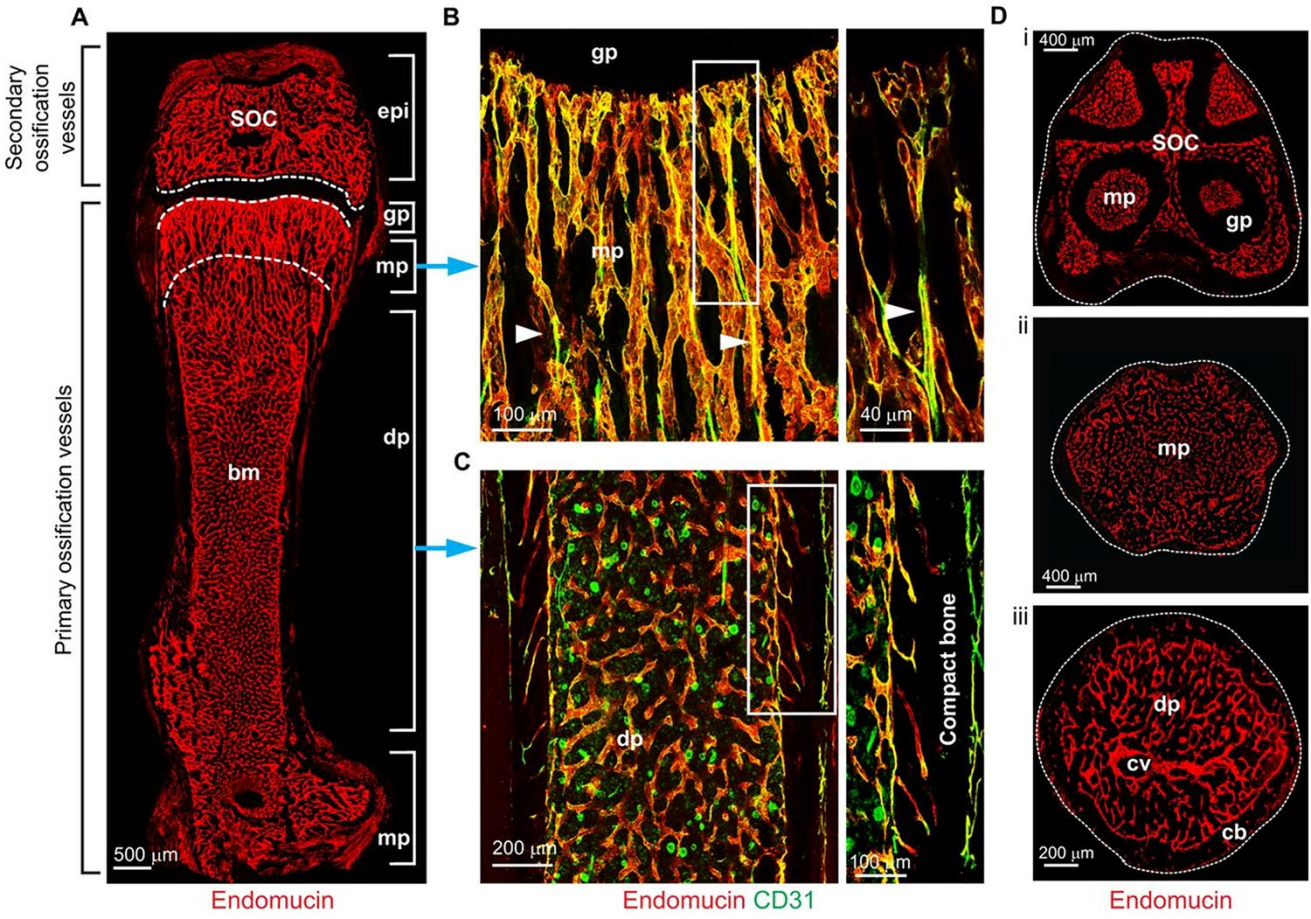
Architecture of the long bone vasculature according to Sivaraj et al. [22]. **A** Confocal image of endomucin (Emcn)-immunostained (red) endothelium in a 100 μm-thick section of P21 murine femur. Regional differences in the organization of the vasculature are evident, as highlighted in the higher magnification images **(B, C)** of the regions marked by blue arrows. **B** In the metaphysis, type H vessels (CD31^high^ Emcn^high^) exhibit a columnar organization and arterial connections (arrowheads); the panel on the right shows a higher magnification of the boxed region. **C** In the diaphysis, highly branched sinusoidal type L capillaries (CD31^low^ Emcn^low^) are found; these connect to endosteal type H vessels in the proximity of compact bone. **D** Confocal images of transverse sections through a P21 femur in the region of the growth plate (i), metaphysis (ii), and diaphysis (iii). *SOC* secondary ossification center, *epi* epiphysis, *bm* bone marrow cavity, *gp* growth plate, *mp* metaphysis, *dp* diaphysis, *cv* central vein, *cb* cortical bone (Reprinted with permission of The Company of Biologists: [Development], [22] copyright (2016))

Similar to other tissues, the vasculature of long bones exhibits a strongly hierarchical architecture with an arterial branch feeding a dense network of capillaries, which drains in venules coalescing into a large vein within the center of the diaphysis [19]. Interestingly, capillaries in bone tissue can be distinguished by their anatomical localization and immunohistochemical markers. Type H capillaries are organized as vessel columns and can be found in the metaphysis, where they express high levels of the junctional protein CD31 and the sialoglycoprotein endomucin (Emcn) [22]. Type L capillaries form a dense, highly branched capillary network in the bone marrow cavity of the metaphysis. These types of capillaries express only low levels of CD31 and Emcn (Fig. 1). Of note, type H and L capillaries are interconnected to a morphological and functional unit, providing a sufficient blood supply for bone development and metabolism [22].

Ten to 15% of the total cardiac output supplies the skeletal vascular system [23], providing the surrounding tissue with adequate amounts of oxygen and nutrients as well as hormones, growth factors and neurotransmitters, such as the brain-derived serotonin [24]. The importance of the bone vasculature into maintaining the bone cells’ survival and activity is illustrated in skeletal diseases, such as craniofacial dysmorphology [25] and idiopathic osteonecrosis [26]. These diseases are caused by insufficient angiogenesis during skeleton development or by an inadequate vascular function within matured bone.

**Fig. 2:**
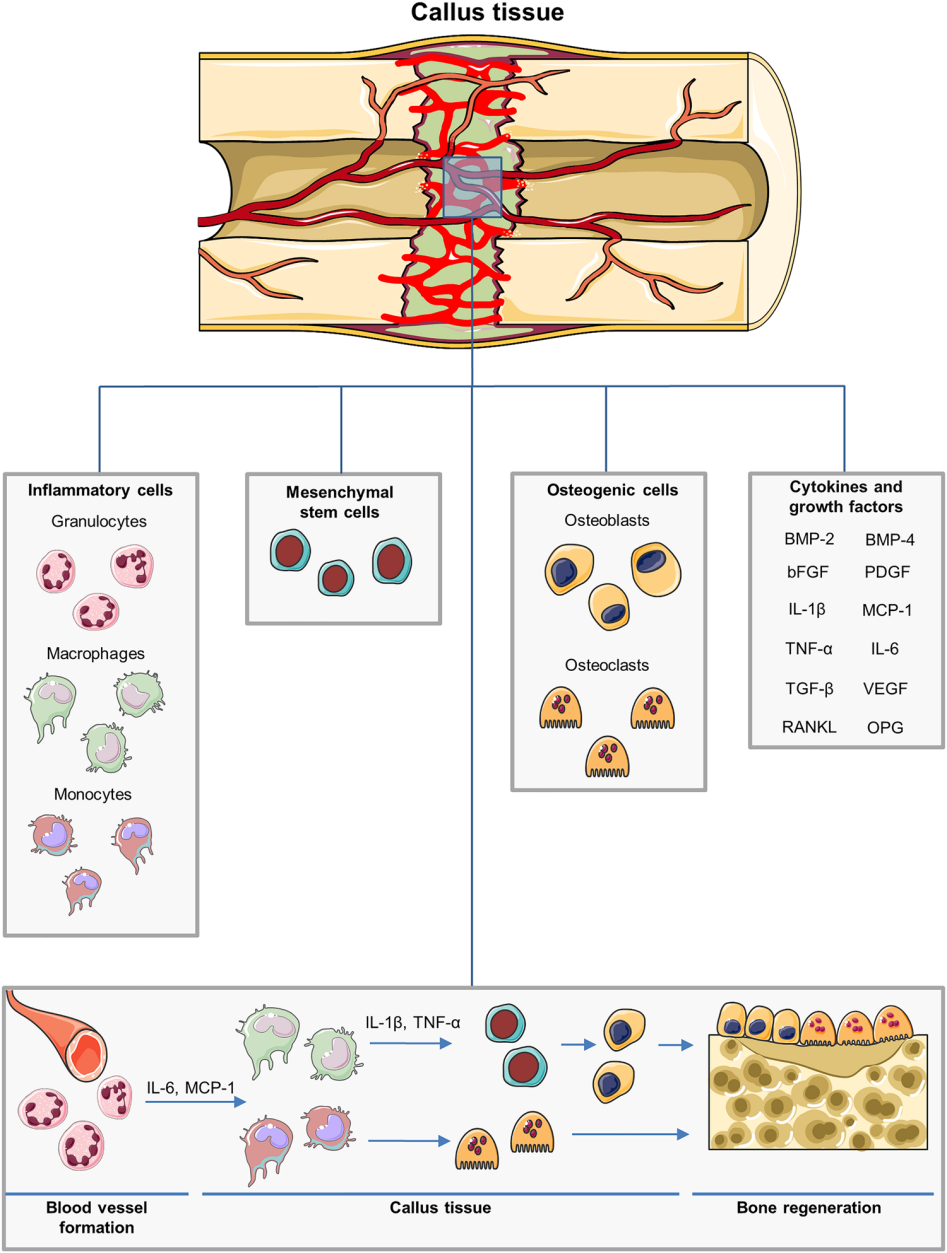
Cell types and growth factors involved in fracture repair. After fracture, a richly vascularized callus tissue is formed, which provides the fracture site with cells vital for bone regeneration. Inflammatory cells, such as macrophages and granulocytes recruit further cell types to the fracture site. MSCs provide a cell pool for differentiation and proliferation. Osteoblasts and osteoclasts coordinate the process of bone growth and remodeling. Moreover, a plenitude of growth factors and mediators are expressed during bone regeneration, including BMP-2 and BMP-4, bFGF, TNF-α, IL-6, IL-1β, TGF-β, monocyte chemotactic protein (MCP)-1, PDGF, RANKL, a stimulator of osteoclastogenesis, OPG, an inhibitor of osteoclastogenesis, as well as the pro-angiogenic factor VEGF. After fracture, the resulting hematoma triggers an immune response. Granulocytes are among the first to arrive at the fracture site by newly formed blood vessels. These cells themselves trigger the migration of macrophages and monocytes to the callus tissue by pro-inflammatory cytokines such as IL-6 and MCP-1. Macrophages initiate the recruitment of MSCs to the fracture site by another repertoire of pro-inflammatory cytokines like IL-1β and TNF-α. Furthermore, MSCs differentiate into osteoblasts, whereas monocytes differentiate into osteoclasts. Subsequently osteoblasts and osteoclasts enable callus remodeling and bone formation at the fracture site

Trauma to the musculoskeletal system induces a disruption of the vital vascular network, resulting in acute hypoxia and necrosis of the surrounding bone tissue [27]. The inflammatory response, which is mainly mediated by macrophages and granulocytes, recruits mesenchymal and osteoprogenitor cells to the fracture site [28, 29] (Fig. 2). These cells infiltrate the callus area through sprouting capillaries originating from the endosteum and bone marrow [12, 30]. Following the establishment of a stable callus tissue within the fracture zone, a remodeling cascade is initiated, in which osteoclastic removal of excessive bone tissue and associated angiogenesis leads to the development of mature lamellar bone [12]. Subsequently, the original bone morphology and the vascular supply is restored. A plenitude of mediators and cytokines is involved in this healing and remodeling process, including bone morphogenetic protein (BMP)-2 and BMP-4 [31, 32], basic fibroblast growth factor (bFGF) [33], transforming growth factor (TGF)-β [34], platelet-derived growth factor (PDGF) [35], receptor activator of NF-κB ligand (RANKL), a stimulator of osteoclastogenesis, osteoprotegerin (OPG), an inhibitor of osteoclastogenesis [36], and vascular endothelial growth factor (VEGF) [37] (Fig. 2). The latter plays a crucial role, not only in the stimulation of angiogenesis during fracture repair, but also in the osteoclast recruitment, activity, and differentiation, and thus, in inducing callus remodeling during the process of endochondral ossification [38, 39].

In addition, in a recent study Romeo et al. [40] identified a novel subtype of vascular associated osteoclasts (VAOs), which are thought to be pivotal for modulating blood vessel growth in bone by directly regulating the anastomosis of type H vessels. Moreover, the authors demonstrated a cartilage resorbing function of endothelial cells that regulates directional bone growth by releasing proteinases such as metalloprotinease-9 [40]. These findings indicate the highly complex cellular interactions between osteogenic and endothelial cells during bone growth and regeneration.

Pericytes are vital for the stabilization and maturation of blood microvessels [41]. Tawonsawatruk et al. [42] demonstrated by injecting pericytes into the fracture site of bone defects in rats that this cell type is capable of preventing non-union formation. In addition, Supakul et al. [43] revealed that pericytes possess the ability to differentiate into osteoblasts and osteoclasts and, thus, directly contribute to the process of bone regeneration.

With a growing elderly population, the aging-associated deterioration of bone regeneration becomes of increasing importance [44]. It has been reported that the impaired fracture healing in the aged is associated with a dysfunction of the bone vascular system, resulting in a delay of angiogenesis during bone repair [44, 45]. Interestingly, aging is associated with a reduction of pericytes within the bone vascular system [46]. Therefore, it may be speculated that the dysfunction of the bone vascular system and the impaired angiogenesis during bone regeneration in the elderly, originates from the age-induced loss of pericytes.

The blood flow within the bone vasculature is thought to play a crucial role for adequate angiogenesis. In a recent study, Ramasamy et al. [47] demonstrated by intravital imaging in mice that a reduced blood flow within the bone vasculature results in an impaired angiogenesis and osteogenesis as well as a downregulation of Notch-signaling of endothelial cells. In aged mice the Notch-signaling activity of endothelial cells is also downregulated, leading to an impaired angiogenesis and osteogenesis [47]. Moreover, the blood flow within the bone vasculature can be severely disturbed by various skeletal and systemic diseases, which then also may lead to alterations of bone regeneration. These include (i) avascular necrosis of the femoral head with a decreased number of endothelial progenitor cells and blood flow interruption caused by a damaged endothelial cell membrane, subsequently resulting in ischemic injury and necrotic cell death, (ii) postmenopausal osteoporosis leading to a decreased blood vessel volume and reduced expression of pro-angiogenic markers, (iii) diabetes mellitus with an associated microangiopathy, causing vasoconstriction and a decreased blood vessel supply and (iv) atherosclerosis, resulting in oxidized lipid formation, which negatively affects bone mass by increasing anti-osteoblastogenic inflammatory cytokines and decreasing pro-osteoblastogenic Wnt ligands [48].

There is strong evidence that a disturbance in the angiogenic response after skeletal injury leads to detrimental consequences for bone regeneration [10]. Various experimental animal studies indicate that the blockade of vascularization by TNP-470, non-steroidal anti-inflammatory drugs (NSAIDs) or fumagillin, hampers fracture repair and may eventually lead to atrophic non-union formation [49–52]. Accordingly, major efforts have been undertaken to establish and validate novel vascularization strategies for the prevention of fracture healing failure. Biophysical stimulation represents a minimally invasive approach to stimulate regenerative and anabolic tissue activities. Applications, such as extracorporeal shock wave therapy (ESWT) [53], low-intensity pulsed ultrasound (LIPUS) [54], low frequency pulsed electromagnetic fields (ELF-PEMFs) [55], and hyperbaric oxygenation (HBO) [56] are able to stimulate the upregulation of pro-angiogenic growth factors, and thus, the process of vascularization, osteogenesis, and bone formation [57–59]. Systemic pharmacological treatment represents another approach, which is feasible and easy to perform in a clinical setting. Erythropoietin (EPO), the primary regulator of erythropoiesis, has been demonstrated in a non-union mouse model to stimulate endochondral ossification and fracture repair [60, 61] by promoting cell proliferation, angiogenesis and bone formation [62]. The parathyroid fragment PTH 1-34 (teriparatide), the main regulator of calcium metabolism, is an additional promising compound to stimulate vascularization and bone regeneration. Teriparatide does not only enhance the migration of pro-angiogenic C45^+^/CD34^+^ cells and the upregulation of VEGF-A mRNA, resulting in an increased neovascularization and cell survival [63], but also accelerates fracture healing [64] and bone formation in segmental bone defects [65]. Moreover, advanced tissue engineering approaches for bone regeneration show great potential in preclinical trials. These strategies have used combined cell populations of pro-angiogenic and pro-osteogenic cell lines, such as endothelial and osteoblastic cells [66, 67], to support bone regeneration in critical bone healing. Notably, the highly vascularized periosteum represents a vital prerequisite for successful fracture repair by providing the cortical blood supply [68] and serving as a source of osteogenic cells [69]. Accordingly, a plentitude of tissue engineering approaches focuses on the design of artificial periosteal substitutes. These tissue engineered constructs consist of a variety of materials including synthetic polymers [70], ceramics [71], and polysaccharides [72]. The addition of cells sheets with mesenchymal stem cells (MSCs) and endothelial cells, which mimic the physiological architecture of the native periosteum, has been used to further stimulate the angiogenic capacity of these periosteal substitutes, thus, showing great potential in promoting vascularization and fracture healing in experimental studies [70, 72].

### The vascularization paradox

**Fig. 3:**
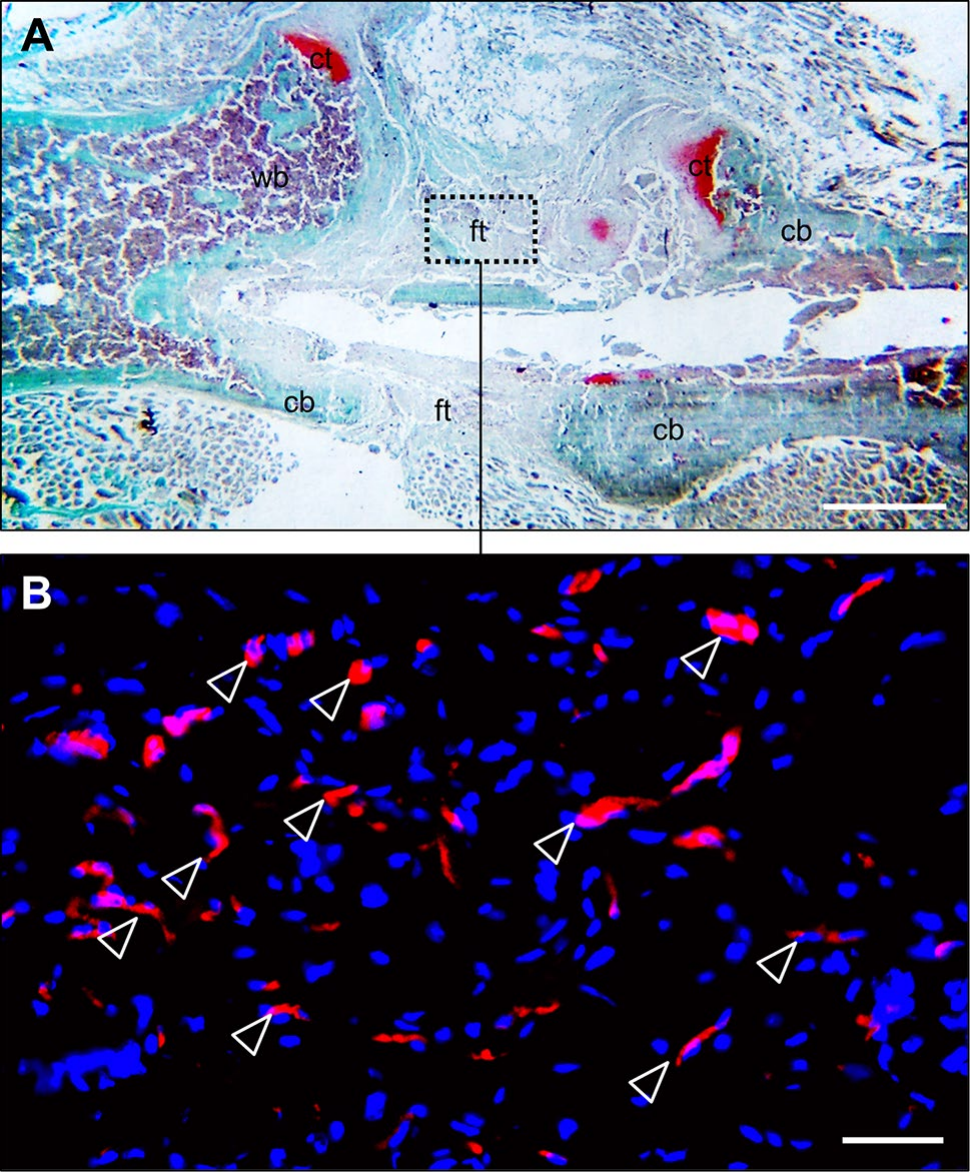
Histological and immunohistochemical image of a non-union in the mouse femur. **A** Safranin-O staining of the callus tissue in a non-union 10 weeks after surgery according to the model described by Garcia et al. [78]. Notably, the fracture gap is filled with fibrous tissue (ft), a typical sign of atrophic non-union formation. Additionally, cartilaginous tissue (ct), woven bone (wb), and cortical bone (cb) are indicated. Scale bar: 1 mm. **B** Immunohistochemical staining of CD31-positive microvessels (red) within the callus tissue in (**A**) (borders marked by dotted line). Cell nuclei are stained with Hoechst 33342 (blue). White arrowheads indicate abundant microvessel formation within the fibrous callus tissue at the defect site. Scale bar: 50 μm

A variety of reviews emphasize the crucial role of vascularization for successful bone regeneration, however till now, no review has delineated the fact that too much vascularization may not improve fracture healing, but may even promote healing failure. Santavirta et al. [73] already reported in 1992 that delayed unions and non-unions consist of vascularized connective tissue. Several other histological studies confirmed these results and could demonstrate that non-unions are indeed considerably vascularized [74–77]. For instance, Garcia et al. [78] analyzed the fibrous callus tissue in a non-union model in mice by immunohistochemistry and detected abundant blood vessel formation within the fracture gap near the cortical bone ends (Fig. 3). In a follow-up study [79], the expression of VEGF, BMP-2, and BMP-4 was additionally analyzed in non-unions by Western blot analyses. Noteworthy, the intrinsic angiogenic response was sufficient for adequate vascularization during non-union formation, however, the failure of fracture healing was associated with a decreased expression of the pro-osteogenic proteins BMP-2 and BMP-4 [79]. Moreover, our own recent investigation on the effects of pantoprazole on fracture healing in aged mice demonstrated that impaired bone healing is associated with a decrease in the protein expression ratio of pro-osteogenic to pro-angiogenic growth factors, such as VEGF and cysteine rich protein(CYR)61 [80].

Eckardt et al. [81] demonstrated that the delivery of VEGF to the osteotomy gap during distraction osteogenesis could not improve blood flow, biomechanical stiffness, and bone formation of the bone regenerate. Interestingly, also the application of the VEGF inhibitor VEGF R2/F Chimera did not affect the process of bone healing [81]. These results may suggest that there are either other factors than VEGF-dependent angiogenesis, which are pivotal for successful bone regeneration, or that angiogenesis during fracture repair is not primarily regulated by VEGF, but other growth factors including BMP-7 [82] and bFGF [83]. In line with these findings, experimental studies by Peng et al. [84, 85] and Chu et al. [86] indicate that VEGF alone is not capable of initiating the cascade of bone regeneration and an overexpression of VEGF can even impair the fracture healing process [84, 85]. Accordingly, clinical studies by Sarahrudi et al. [87] and Weiss et al. [88] demonstrated that serum levels of VEGF are increased in patients with non-unions when compared to patients with successfully healed fractures. However, it is unclear, if the overexpression of VEGF leads to non-union formation or if the non-union formation causes its compensatory overexpression. One possible cause for the latter may be the hypoxic conditions within the callus tissue of non-unions due to an impaired functionality of the vascular network. The detection of endothelial cells within the callus tissue of non-unions is not necessarily associated with a functional vascular network, but can also represent regressing vascular structures without blood perfusion. This may also explain the reduced capacity for osteogenic proliferation and differentiation at the fracture site, because cell survival is of paramount importance under hypoxic conditions. Furthermore, the degraded vascular network may decrease the clearance of VEGF within the callus tissue. Because the control of VEGF clearance is known as crucial mechanisms to regulate VEGF activity [89], this may explain the increased VEGF levels observed in non-unions [79]. Finally, it should be considered that successful bone regeneration depends on the temporal and spatial expression patterns of growth factors [90] as well as on the ratio of pro-osteogenic to pro-angiogenic growth factors within the callus tissue, in particular the ratio of VEGF to BMP-2 and BMP-4 [79, 84, 85].

Despite the research progress during the last two decades, no ideal management for the prevention and treatment of non-union formation could be introduced into clinical practice so far. This may be due to the fact that vascularization is only one of many important factors, which are required for bone regeneration. Noteworthy, non-unions exhibit a decreased pool and delayed proliferation of MSCs as well as altered serum levels of related chemokines and growth factors, such as leptin, interleukin-6 (IL-6), platelet-derived growth factor-BB (PDGF-BB), stem cell factor (SCF), and insulin-like growth factor (IGF-1) [91]. However, the number of early and late outgrowth endothelial progenitor cells (EPCs) and their regulating pro-vasculogenic growth factors, such as angiopoietin (Ang)-1, Ang-2, stromal-derived factor-1 (SDF-1), interleukin (IL)-8, VEGF, transforming growth factor-β-1(TGF-β-1), and Dickkopf-related protein-1 (DKK-1) are not significantly affected in non-union patients [91]. These findings indicate that non-unions are, in fact, not associated with vascular degeneration and maintain the ability for both, the generation of novel blood vessels as well as angiogenesis by paracrine mechanisms. Furthermore, they emphasize the role of a mesenchymal and osteogenic cell pool defect and their related growth factors in the pathogenesis of non-union formation. Hence, it may be assumed that treatment strategies for non-unions should rather focus on the stimulation of osteogenesis than angiogenesis. This hypothesis is supported by experimental studies, which analyzed the effect of BMP-2 and VEGF co-delivery for bone regeneration. Although the sole delivery of VEGF demonstrated a stimulation of blood vessel formation, no significant increase in bone formation was detected [92–96]. In contrast, Uhrig et al. [97] could demonstrate that a transient ischemic insult and a subsequent recovery response significantly enhance BMP-2-mediated bone defect repair. This highlights the complexity of the relationship between vascularization and bone regeneration [97]. Clinical studies investigating the effect of surgical angiogenesis by the generation of arteriovenous bundles and vascularized bone grafts did not report an improved bone viability and union rate [98–100]. Although, it is well accepted in clinical practice that large bone defects of more than 6 cm should be supplied by vascularized bone grafts (VBGs), Allsopp et al. [99] could not confirm that VBGs are superior to non-vascularized ones (NVBGs). Accordingly, a clinical study by Schuh et al. [101] with a mean follow-up of 52 months demonstrated in diaphyseal bone reconstruction that NVBGs result in a similar radiographic and clinical outcome compared to VBGs. Moreover, NVBGs compared to VBGs tend to a lower rate of complications and revision surgery, which are mostly due to problems with wound healing related to the use of myocutaneous flaps for vascular bone grafts [101].

Even more astonishing are the findings of Orth et al. [102], who implanted hydrogels loaded with adipose tissue-derived microvascular fragments (MVF) in murine femur defects to improve vascularization and, thus, bone regeneration. However, the data of this study shows that these highly angiogenic vascularization units did not improve but even impaired the formation of new bone within the defects [102]. These findings are supported by an experimental studies of Ruehle et al. [103, 104], which evaluated the effects of BMP-2 compared to BMP-2 in combination with MVFs in bone defects with concomitant muscle loss. The results showed a decreased bending stiffness and larger areas of non-mineralized, marrow-like tissue in bone defects additionally treated with MVFs [103]. These observations imply that extensive angiogenesis and vascularization may not support, but, paradoxically, may even hamper adequate fracture repair and, therefore, aggravate non-union formation.

## Future perspectives

In the future, novel imaging technologies, such as multi-photon fluorescence microscopy [105, 106] and photoacoustic imaging [107, 108], may markedly improve our knowledge of the functionality of the microvascular networks in non-unions by the direct measurement of oxygen saturation within the callus tissue. In combination with advanced immunohistochemical staining methods and molecular biological approaches it may be possible to identify and investigate potential growth factors and mediators involved in the pathology of vascular dysfunction during non-union formation. Furthermore, the development of sophisticated multiscale simulation models, which allow to assess the influence of angiogenesis and oxygenation on fracture healing [109–111], may help to fully understand the mechanisms of failed fracture healing and to simulate the effectiveness of specific treatment strategies. Thus, emerging treatment approaches may be able to specifically improve the functionality of these microvascular networks. Moreover, emerging treatment strategies should not only consider the stimulation of angiogenesis as a key target for bone regeneration. In fact, other factors than vascularization may substantially contribute to successful fracture repair, including mechanical stability, patients’ physiological state and comorbidities as well as the availability of pro-osteogenic mediators and cells at the fracture site [1]. Thus, emerging treatment strategies should also consider these factors to improve bone regeneration. If this succeeds, also challenging cases of non-unions in clinical practice may successfully be treated and cured.

## Conclusion

**Fig. 4:**
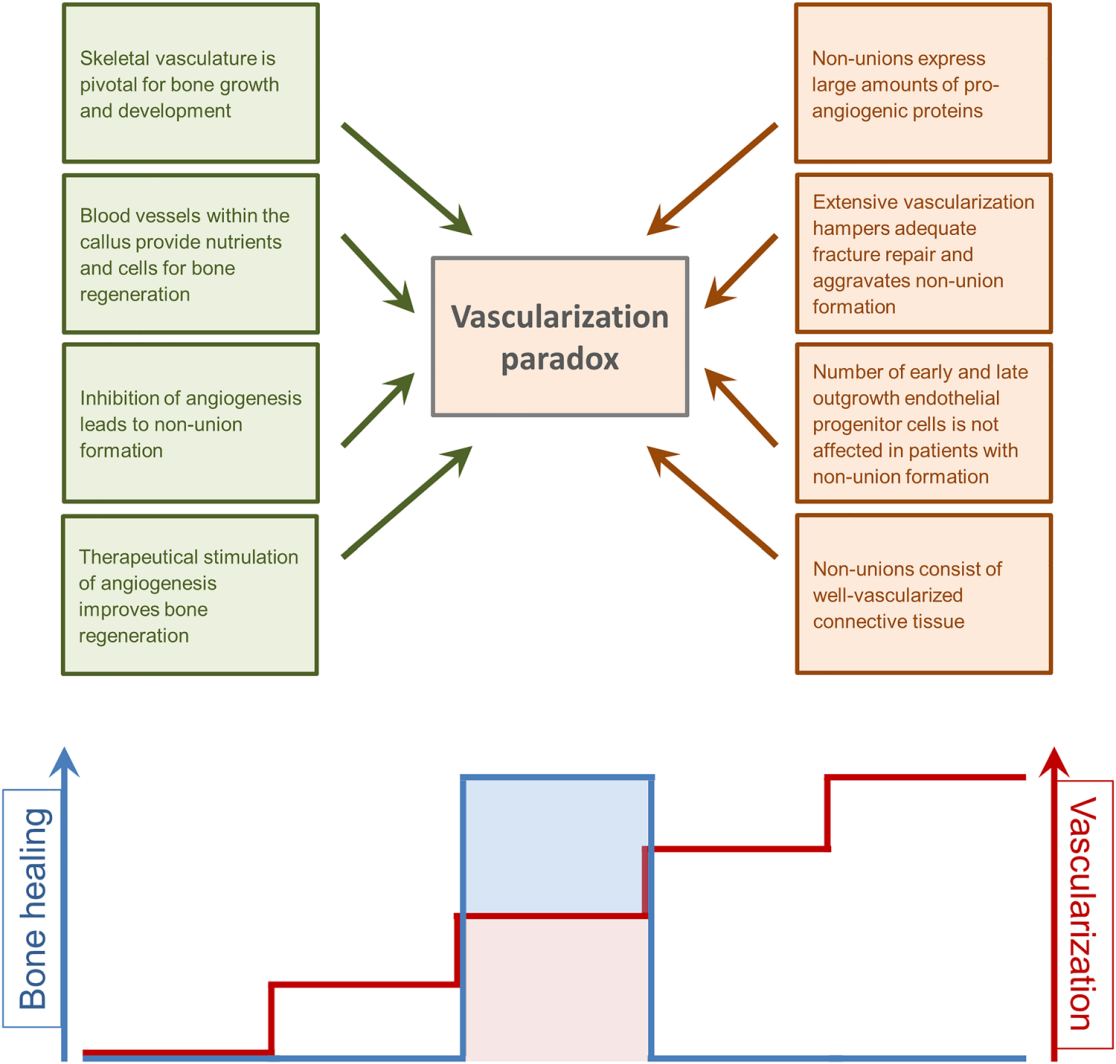
Illustration of the conflicting arguments of the “vascularization paradox”. Angiogenesis and vascularization are pivotal for bone growth and development as well as for the supply of nutrients and cells at the fracture site. Moreover, inhibition of angiogenesis hampers fracture repair, whereas stimulation of angiogenesis improves bone regeneration. On the other hand, non-unions demonstrate a sufficient expression of pro-angiogenic proteins. Moreover, experimental studies have demonstrated that extensive angiogenesis and vascularization hamper adequate fracture repair and aggravate non-union formation. In addition, patients with non-union formation show no alteration in the number of early and late outgrowth EPCs. Finally, the callus tissue of non-unions consists of well-vascularized connective tissue. It may be speculated that a reduced as well as an extensive vascularization impairs fracture healing and, thus, leads to non-union formation. A well-balanced temporal and spatial angiogenesis within the callus tissue, however, promotes fracture repair, resulting in successful bone healing

Although, it is well accepted that the deterioration of angiogenesis and vascularization is a crucial factor for the failure of fracture healing, there is increasing evidence that this hypothesis is oversimplified. In fact, multiple studies demonstrate a considerable vascularization and even overexpression of pro-angiogenic factors within the callus tissue of non-unions. Of interest the application of highly angiogenic vascularization units in large bone defects did not improve bone formation, but, paradoxically, aggravated non-union formation [102]. This may represent a vascularization paradox in non-union formation (Fig. 4). Thus, the role of vascularization in non-union formation still remains to be determined.
